# Low Vision Aids in Glaucoma

**DOI:** 10.5005/jp-journals-10008-1104

**Published:** 2012-10-16

**Authors:** Anjani Khanna, Parul Ichhpujani

**Affiliations:** 1 Glaucoma Services, Government Medical College and Hospital, Chandigarh, India; 2 Glaucoma Services, Government Medical College and Hospital, Chandigarh, India

**Keywords:** Magnifier, Rehabilitation, Telescope, Visual field loss.

## Abstract

A large number of glaucoma patients suffer from vision impairments that qualify as low vision. Additional difficulties associated with low vision include problems with glare, lighting, and contrast, which can make daily activities extremely challenging. This article elaborates on how low vision aids can help with various tasks that visually impaired glaucoma patients need to do each day, to take care of themselves and to lead an independent life.

## DEFINING THE PROBLEM

Before we proceed further, let’s understand the gamut of terminology related to qualitative and quantitative visual acuity.

### Functional Vision

This refers to the use of vision for a particular activity. Even small amounts of vision can be helpful, for example to recognize a person closeup, or to avoid stumbling on objects. How an individual utilizes his vision depends on his/her experiences and can vary depending upon the severity of their condition. Functional vision may be improved with refractive correction, low vision devices or guidance as regards the use of vision.

### Functional Vision Impairment

This refers to significant limitation of visual capability, which is manifested by insufficient visual resolution, restricted field of vision and reduced contrast sensitivity. Functional impairment causes difficulty in performing tasks or daily activities.

### Economic Blindness

This is defined as visual performance with a distance visual acuity of 6/60 or less in the better eye with best correction or as a defect in the visual field so that the widest diameter of vision subtends an angle no greater than 20°.

### Legal Blindness

It is the legal definition used to determine whether an individual with vision impairment is eligible for government benefits. It is 6/60 or less with best correction or a visual field of 20° or less in the widest meridian of the better eye.

### Low Vision

A person with low vision has impairment of visual function even after treatment and/or refractive correction, and has a visual acuity in the better eye <6/18 to light perception (LP), or a visual field of <10° from the point of fixation but who uses or is potentially able to use vision for the planning or execution of the task. This low vision definition however does not include standards of near vision, which is the main area dealt with low vision patients.

## MAGNITUDE OF THE PROBLEM

According to a WHO survey glaucoma is emerging as the second leading cause of blindness worldwide. Recent studies have shown that there will be 60.5 million people with open angle glaucoma (OAG) and angle closure glaucoma (ACG) in 2010 increasing to 79.6 million by 2020.^1^ Bilateral blindness will be present in 4.5 million people with OAG and 3.9 million with ACG in 2010 rising to 5.9 and 5.3 million in 2020 respectively.^2^

The true magnitude of the low vision in glaucoma is not known mainly because of no standard protocol and uniform definition of low vision, incomplete surveys and statistical classification of eye diseases across the world. The risk of glaucoma patients becoming visually handicapped is seen to be 11% and becoming blind from glaucoma is 0.7 to 1.9%.^3^

## WHAT ARE THE VISUAL FIELD AND ACUITY REQUIREMENTS FOR DAY TO DAY ACTIVITIES?

The peripheral limits of normal visual fields (with maximum target stimulation) are approximately 60° above and nasal, 70 to 75° below and 100 to 110° temporal to fixation.

For routine activities like driving a minimum visual acuity of 20/200 with 120° of visual field (for daytime driving at 40 mph) is required and for fluent reading with a print size and contrast several folds than the threshold a minimum visual field of four letters is required.

## WHAT GOES WRONG IN GLAUCOMA?

In the leading causes of low vision like age related macular degeneration, glaucoma, diabetic retinopathy, retinitis pigmentosa, central retinal vein occlusion (CRVO), corneal damage, etc. the visual field loss patterns are characteristic in some disorders like restriction of peripheral vision with sparing of central fields in early disease in glaucoma, progressive concentric loss, complete or incomplete midperipheral ring scotoma with residual central field associated with small peripheral island in retinitis pigmentosa, etc.

## HOW WE CAN HELP?

### Vision Rehabilitation

Rehabilitative services provided to both who are partially sighted and those who are blind. These services include mobility training, adaptive skills training, low vision instruction career services and training, psychological counseling and others.

Common needs of such patients include:

- Recognizing faces and objects
- Reading or writing, signing documents, etc.
- Distance or TV watching disabilities
- Adapting to changes in the different levels of light, especially at night
- Adjusting to glare: Outdoors, indoors or both?
- Mobility in unfamiliar areas
- Depth perception

Low Vision Aids

Low vision aids are devices which help people use their sight to better advantage. These aids can be optical lenses, such as magnifiers or telescopes or nonoptical devices, such as visors, filters, reading slits, stands, lamps and large print.

*Basic principle:* The basic principle of all low vision optical devices is to magnify. The specified magnifying powers of most of the available aids are computed from the single formula:

Magnification (M) = Dioptric power (D)/4

This formula works on the assumption that with the unaided eye the patient can sustain just enough accommodation to hold the object at 25 cm. It also assumes that when magnification is used, the reading material is placed at principal focal plane of the lens (neither of these assumptions is practically true in practice).

Thus, magnification can be varied by changing the distance from the object to lens. This group includes spectacles and hand magnifiers.

The formula can thus be written as M = D + A/2.5, where A is the amplitude of accommodation.

If the distance between eye and lens is appreciable then magnification is given by the formula:

M = D + A - h AD/2.5, where h is the eye lens distance in meters.

### Low Vision Aid Work-up

A battery of tests need to be done to evaluate patient’s visual function, including visual acuity, visual fields (central and peripheral), contrast sensitivity and color vision. The information obtained from this testing will provide clues about whether devices may be beneficial and, if so, what types of technology to prescribe?

It is important to ask the patient what activities he/she does on a regular basis. Knowing patients’ literacy levels are also very important. Illiterate patients have no concern about reading or writing.

### Optical Devices

**Figs 1A to C:**
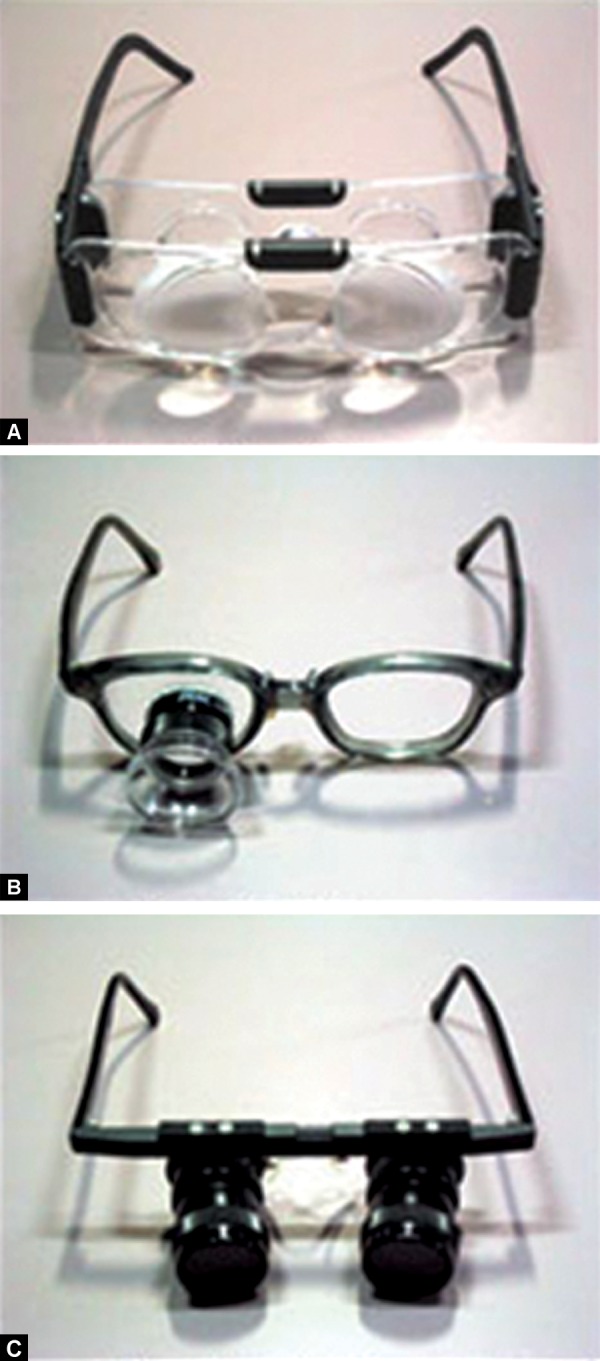
Reading devices: (A) Magnifying glasses, (B) mounted loupes, (C) reading telescopes (source: *www.lowvision.org)*

**Figs 2A to C:**
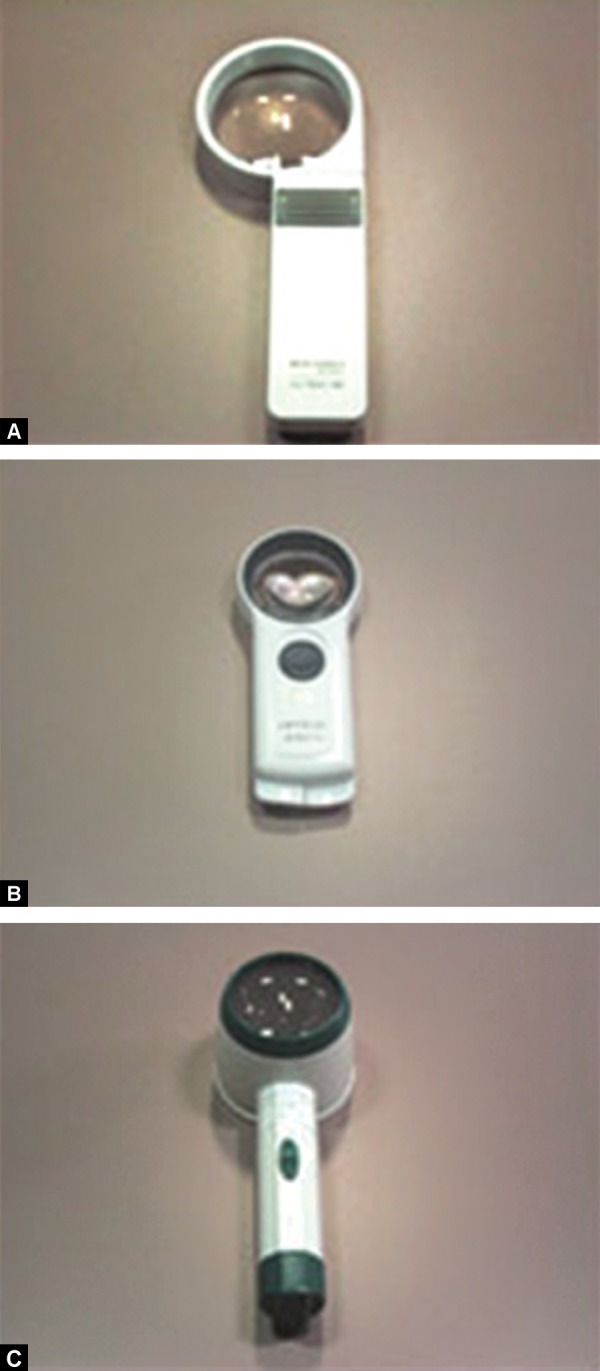
Magnifiers: (A) Illuminated hand magnifier, (B) hand magnifier, (C) illuminated stand magnifier (source: *www.lowvision.org)*

**Figs 3A to C:**
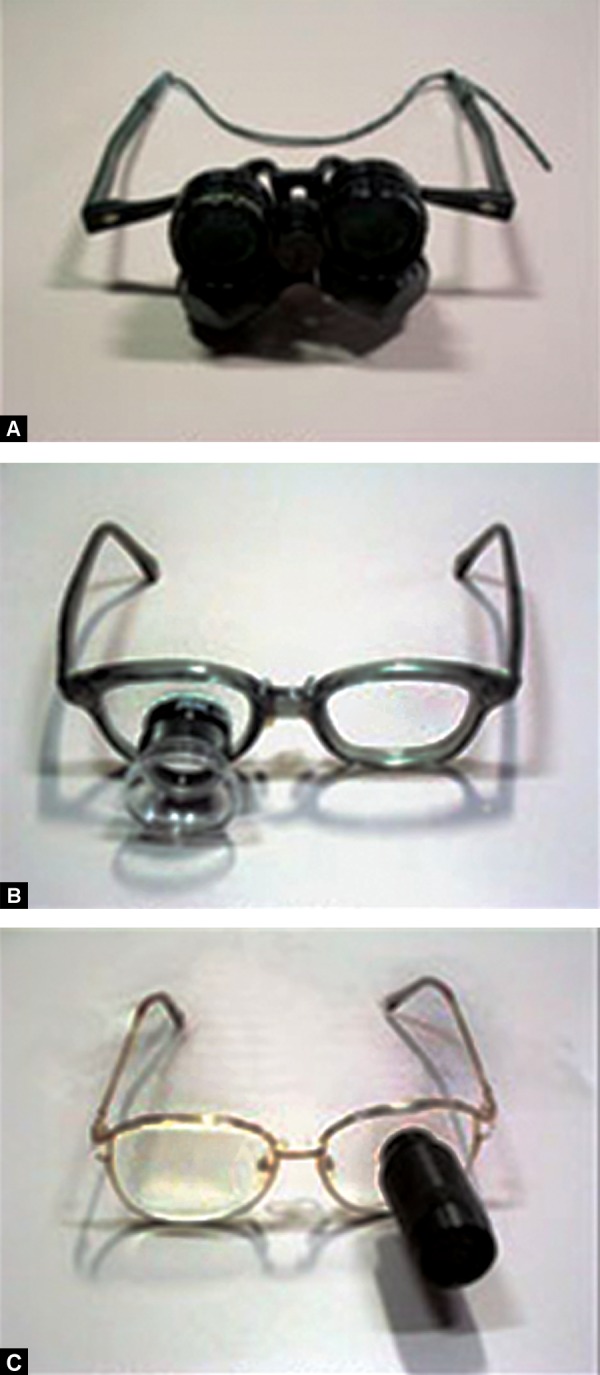
Distance viewing devices: (A) Binocular telescopes, (B) telescopes, (C) mounted telescopes (source: *www.lowvision.org)*

1. Magnifying spectacles (high plus reading glasses) used for reading any material, writing and looking at objects from close range. The spectacle produces magnification of 1/4th of the power of the lens (Figs 1A to C). For binocular corrections prism spectacles half eyed of full field with base in (to compensate for convergence angle ofthe eye) are used.
2. *Magnifiers:* Handheld low vision magnifiers are helpful for looking in a mirror, telling the time on a watch, and other quick viewing tasks. The ones with self-contained illumination can be used when surrounding illumination is dim. The low vision magnifiers that are mounted on a stand are great for reading books and doing close-up work such as needlepoint and quilting (Figs 2A to C).
3. *Telescopes:* These are prescribed for distance, near and intermediate tasks like reading signs, recognizing people, reading from blackboard > 2 m, watching television, games or traffic signals. These can be hand held monocular, clip on, spectacle mounted, monocular or binocular, bioptic designs (Figs 3A to C).

### Nonoptical Devices

To enhance the images and to reduce glare typoscopes are used. Felt tipped pens, bold lined papers, writing guide, large print materials and adequate lighting on print are helpful methods in assisting vision by enhancing contrast while reading stands provide a comfortable working distance.

### Assistive Technologies

**Figs 4A to D:**
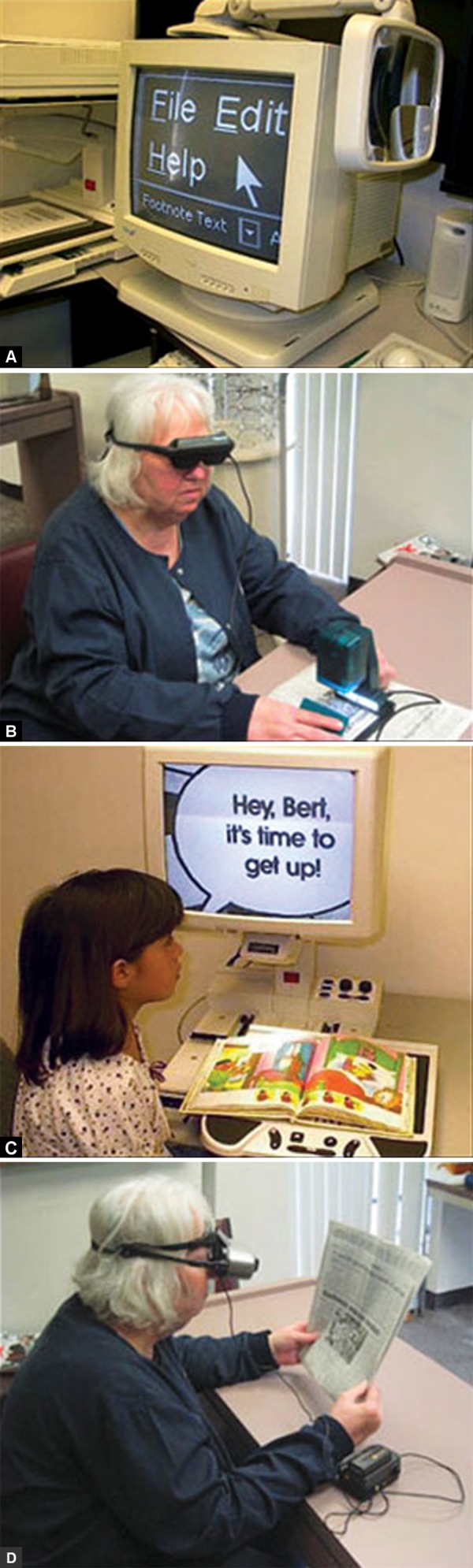
(A) Computer magnification software, (B) patient using a hand-held camera, (C) patient using a CCTV, (D) patient using a head-mounted camera (source: *www.lowvision.org*)

Desktop electronic magnifiers are low vision aids that display reading material that is placed on a tray. The person with low vision moves the reading material as needed and views it on a screen at eye height, above the tray. Most of these magnifiers have a monitor, but others allow for connection to a TV or computer monitor. Often these devices are a bit heavy and therefore are impractical to take to a different location at will. Portable low vision electronic magnifiers come in two main types. The handheld ones can usually be carried in a pocket or handbag, and are used to read labels in a grocery store or pharmacy, menus in a restaurant, credit card slips, price tags and more. They are meant for quick reading tasks, and the newer models even have a camera that takes a still image of the reading material so it can be viewed either later or at a more convenient angle. Another example of an electronic low vision magnifier is a camera that is mounted on an adjustable stand. It can be aimed near or far, so the person with low vision can view himself, reading material on a table, a distant blackboard, or a TV that is across the room, on a monitor that is attached to the camera (Figs 4A to D). Some units can be hooked up to a TV or laptop computer instead of a dedicated monitor. These units may be portable, if they are light enough in weight. Since more people have laptop computers now, many of these electronic low vision aids have been integrated with the computers with special software, so the user can view reading material and objects using the laptop screen, and even use the mouse to control some or all of the display settings.

Assistive devices for daily living like talking scales, color identifiers, talking compasses, etc.

### Low Vision Aids in Glaucoma: Scientific Evidence

Hadded et al showed the benefit of low vision aids and early correction of refractive errors in children with congenital glaucoma improving the global development of the children.^4^

Khan et al in their study on 410 patients from their low vision clinic of the age group 11 to 20 years provided standard prescription spectacles to 174 patients.^5^ Handheld distance telescopes were prescribed for board work for 45 students who had best corrected visual acuity ranging from 2/60 to 6/30 in the better eye all of whom were able to achieve visual acuity of> 6/18, whereas almost 85% had a vision of > 6/12 with the prescribed telescopes. The spectacle magnifiers for reading and writing tasks were prescribed to 187 patients who presented with a reading acuity of N48 to N12. Nearly half these patients (49.2%) improved to N6 and 39% to N8, and the rest to N10. Fourteen patients with reading acuity from N24 to N12 were prescribed bifocals. All achieved a reading acuity of > N8, and 64.3% had N6 vision. Nine patients who had reading acuity ranging from N36 to N12 were given hand/stand magnifiers; these patients achieved N12 or better vision. Among these 55.6% achieved visual acuity of N6, one improved to N12 and three improved to N10 or N8. Three patients with reading acuity of N42 were provided with closed-circuit television (CCTV), and showed improvement to N6 reading acuity. Nonoptical aids enhanced subjectively the use of vision in these patients with or without the optical devices.

Silva and colleagues^6^ found that the most frequent etiology for low vision in infants and adolescents was congenital glaucoma and in patients aged between 20 and 39 years were ocular toxoplasmosis (21.1%). In patients with 40 to 59 years old, pigmentary retinosis was the most frequently pathology (19%). In elderly people, it was glaucoma (49%). The telescopic system was the only optical aid indicated for distance (44%) and glasses were the most indicated for near (54.5%).

Haddad et al analyzed data from 100 children with congenital glaucoma and found that 2% had normal visual acuity levels, 29% mild visual impairment, 28% moderate visual impairment 15% severe visual impairment, 11% profound visual impairment and 15% near blindness.^4^ Sixty-eight percent received optical correction for refractive errors. Optical low vision aids were adopted for distance vision in 34% of the patients and for near vision in 6%. A manual monocular telescopic system with 2.8x magnification was the most frequently prescribed low vision aid for distance, and for near vision a +38 diopter illuminated stand magnifier was most frequently prescribed.

Therefore, effective low vision intervention in patients with advanced visual field damage due to glaucoma should start as soon as the patient experiences difficulty performing day-today tasks. Realistic goals of intervention and the devices that could be helpful for the patient should be discussed with the patient. Since the visual acuity, visual field loss and contrast sensitivity progressively deteriorate with advancing age, the sooner patients adopt low vision aid devices, the better it is.
